# Full-order adaptive sliding mode control with extended state observer for high-speed PMSM speed regulation

**DOI:** 10.1038/s41598-023-33455-x

**Published:** 2023-04-17

**Authors:** Meng Luo, Zhichun Yu, Yang Xiao, Liyong Xiong, Qiang Xu, Li Ma, Zhihong Wu

**Affiliations:** 1 Intron Technology (Shanghai) Co., Ltd., Shanghai, 201203 China; 2 School of Automotive Studies, Tongji University, Shanghai, 201800 China

**Keywords:** Engineering, Electrical and electronic engineering

## Abstract

In order to achieve speed control of high-speed permanent magnet synchronous motor with high precision, the sliding mode control (SMC) is usually adopted. However, the inherent chattering phenomenon affects the speed control performance. In order to solve this problem, a composite speed regulator is proposed in this paper, which is made up of two parts: the adaptive full-order SMC and extended state observer (ESO). Switching gain adaption law is proposed for minimizing chattering as much as possible while ensuring the robustness of sliding mode control. The total disturbance is estimated by the ESO and through feedforward, thus improving the system anti-disturbance ability. Finally, the effectiveness of the proposed speed regulator has been validated in the test bench.

## Introduction

The high-speed permanent magnet synchronous motor (PMSM) plays an important role for automotive applications due to its high performance, efficiency and power density^1^. In the speed control, the drive systems will be affected by the change of internal parameters (such as the change of output torque caused by the change of rotational inertia and inductance), and the change of external load torque of the system. In order to improve the robustness of speed loop in more high-performance applications, there have been many researches.

PI control is simple and easy to implement, which is the mainstream control method of speed loop^1^. Although one-degree-of-freedom (1DOF) PI controller can handle the disturbance rejection, but it’s very limited, since there exists contradiction between overshoot and disturbance rejection performance. Moreover, due to the zero introduced by the PI controller, the overshoot of the speed loop is unavoidable. This problem can be handled by replacing it with IP controller, but the bandwidth of that structure is lower than PI controller^2^. To resolve the contradiction between overshoot and disturbance rejection, two-degree-of-freedom (2DOF) PI controllers are exploited^3–5^. In Ref.^3^, the 2DOF PI controllers are designed considering the loop transfer function, taking the reference command into consideration. Those controllers can handle the tracking and anti-disturbance respectively. In Ref.^5^, a new 2DOF PI control speed controller is proposed, which can not only ensure the speed tracking performance, but also effectively improve the anti-load disturbance ability of the system and improve the robustness of the system to the change of inertia. However the structure is complex compared with the 1DOF PI controllers, which means the parameters tunning may be difficult and the anti-windup^6,7^ algorithms are also different.

However, PI control has poor robustness due to torque mutation and parameter variation, resulting in large speed fluctuation. Therefore, improving the robustness of speed loop becomes one of the important indexes to measure the performance of speed loop. Sliding mode control (SMC) is very popular with researchers because of its strong robustness and simple structure^8^. But the existence of chattering is the biggest problem of SMC due to various undesirable factors^9^. The boundary layer theory reduces the chattering problem of the system by changing the switching function into a continuous function, such as: saturation function^10^ or hyperbolic tangent function^11^, but this method can only achieve practical stabilization and the control accuracy of the system. i.e., the steady-state error of the speed, depends on the parameters of the continuous function. In order to eliminate the chattering problem in the given signal of torque command, the improved reaching law is used^12,13^ (i.e., the reaching law is designed by combining the state of the systems), but this greatly reduces the robustness of SMC, reduces the control accuracy of speed controller, and the speed tracking error cannot converge to zero strictly. An adaptive sliding mode controller design method is proposed in Ref.^14^, but the switching control term appeared explicitly in the design of the control law, and no strategy to weaken or eliminate chattering was considered. Therefore, there was high-frequency chattering in the control signal, which affected the stability of the system. High-order SMCs are introduced in the literature to reduce the chattering by taking the discontinuous terms into the integrals, which sufficiently degrades the chattering phenomenon^15,16^. But the complex structure and difficult stability proof are produced. In addition to the improvement of the main speed controller, the disturbance observers^17,18^ are introduced to estimate the total or part disturbance which will be feed forward to the controller output. Due to the compensation for the disturbance, the speed controller can handle the disturbance rejection and the dynamic of the system will be enhanced. If the compensation is feed forward to the SMC, the switching gain determined by the total disturbance will be reduced and hence, the chattering will be degraded too.

In view of the above analysis, in order to further reduce the chattering phenomenon of the sliding mode control system and maintain the simple structure of the controller as far as possible, a composite speed regulator is proposed, which is made up of two parts: the adaptive full-order SMC and the extend state observer (ESO). The main contributions include:

1. Adaptive full-order SMC is introduced, which maintains the simple structure of first-order SMC. The discontinuous terms are hidden in the integral and thus, chattering reduction is achieved.
2. Switching gain adaption law is given for minimizing chattering as much as possible while ensuring the robustness of sliding mode control. The total disturbance is estimated by the ESO and through feedforward, and the compensation is added in the controller output to improve the system anti-disturbance ability.

This paper is structured as follows. The mechanical model of PMSM is introduced in “Mechanical model of PMSM” at length. In “Composite speed controller design for high speed PMSM”, the proposed composite speed regulator will be discussed compared with other traditional speed regulators. Comparative experiment are carried out to prove the superior performance of the proposed speed regulator. Finally, the “Experimental verification” will conclude the paper.

## Mechanical model of PMSM

The speed of the motor is determined by the torque, friction, load and the moment of inertia of the system. The traditional mechanical model of PMSM can be described as follows by Newton’s law of the rotating rigid bodies:

1 $$\left\{ {\begin{array}{*{20}c} {\dot{\omega }_{m} = \frac{{T_{e} }}{J} - \frac{B}{J}\omega_{m} - \frac{{T_{L} }}{J} {\mkern 1mu} {\mkern 1mu} } \\ {d(t) = - \frac{B}{J}\omega - \frac{{T_{L} }}{J} - \frac{1}{{J_{n} }}T_{e}^{*} + \frac{{T_{e} }}{J},} \\ \end{array} } \right.$$

where $\omega_{m}$ is the mechanical speed of the motor, $T_{e}$ is the actual electromagnetic torque generated by the motor and $T_{e}^{*}$ is the command electromagnetic torque generated by the speed control. The difference between them takes into account the current or torque loop dynamics and the digital signal processing. $T_{L}$ is the load torque and $B$ is the viscous friction coefficient. $J$, $J_{n}$ are the actual moment of inertia and nominal moment of inertia used for speed control. $d(t)$ denotes the total disturbance. Considering the above inner and external disturbance, the model for designing the speed controller is shown in (2):

2 $$\dot{\omega }_{m} = J_{n}^{ - 1} T_{e}^{*} + d(t).$$

From a practical point of view, $d(t)$ is bounded and changes slowly with time, which means $|d(t)| < \sigma$ and $|\dot{d}(t)| \le \, \Delta$. High speed air compressor system is affected by various disturbances such as airflow disturbance, unmodeled variations and uncertainties, and have nonlinear characteristics. The high-precision speed regulation should fully consider the complex disturbances and uncertain factors, which can be realized by the composite speed controller established in this paper.

## Composite speed controller design for high speed PMSM

In this section, we will discuss the drawback of the traditional SMC, firstly. To mitigate the chattering problem, three parts are introduced: full-order SMC, the switching gain adaptive law, and the disturbance observer. In addition, the stability of the speed loop is guaranteed by the Lyapunov stability theory.

### Traditonal SMC

The design of sliding mode controller is divided into two parts: one is the selection of sliding mode surface; the second is the design of sliding mode controller. The sliding mode surface is selected so that the system can operate in a desired way after reaching the sliding mode surface, and the controller is designed to ensure the existence or arrival conditions of the sliding mode, so that the system can reach the sliding mode surface and remain on the sliding mode surface in a limited time^19^.

The speed loop is a first-order system. In order to design the sliding mode control and satisfy the reduced-order dynamics since reaching sliding phase, the sliding variable is set as the error $e$ between the reference speed $\omega_{m}^{*}$ and the measured speed $\omega_{m}$.

3 $$s = e = \omega_{m}^{*} - \omega_{m} .$$

In order to make the control quantity explicit, the derivative of the error can be expressed as:

4 $$\dot{s} = \dot{\omega }_{m}^{*} - \dot{\omega }_{m} = \dot{\omega }_{m}^{*} - J_{n}^{ - 1} T_{e}^{*} - d(t).$$

The exponent reaching law can be used for designing the torque command:

5 $$T_{e}^{*} = J_{n} [\varepsilon \cdot sign(s) + k \cdot s + \dot{\omega }_{m}^{*} ],\varepsilon ,k > 0.$$

The Lyapunov asymptotic stability can be easily derived by selecting $1/2 \cdot s^{2}$ as the alternative Lyapunov function^20,21^.

In order to obtain the robustness during the sliding phase, the switching gain need to be sufficiently large to counteract the effects of lumped disturbances. In the actual situation, it is hard to obtain the disturbance term $d(t)$ in real time, so the switching gain is set larger enough to satisfy $s\dot{s} < 0$. As mentioned in previous section, continuous SMC and SMC based on improved reaching law^8^ can reduce the chattering phenomenon, but those methods only achieve practical stabilization, which reduce the robustness of SMC.

Although traditional SMC has the robustness during the sliding phase, the large switching gain will result in large chattering, which may degrade the control performance and introduce more undesired harmonics to the current loop.

### Full-order SMC

However, according to reference^22^, if the full-order sliding mode controller is designed, the control variable can be designed in the form of integral of the discontinuous control term. Thus, the purpose of suppressing high frequency chattering is achieved. The full-order means that during the sliding phase, the dynamics of the system is not reduced. Therefore, the corresponding sliding variable is designed as:

6 $$s = ce + \dot{e},\quad c > 0.$$

Substituting Eq. (2) into Eq. (6), we obtain:

7 $$\begin{aligned} s & = ce + \dot{e} \\ & = c\left( {\omega_{m}^{*} - \omega_{m} } \right) + \dot{\omega }_{m}^{*} - \dot{\omega }_{m} {\mkern 1mu} {\mkern 1mu} \\ & = c\left( {\omega_{m}^{*} - \omega_{m} } \right) + \dot{\omega }_{m}^{*} - J_{n}^{ - 1} T_{e}^{*} - d(t). \\ \end{aligned}$$

The torque command can be design into two parts:

8 $$T_{e}^{*} = u_{eq} + u_{r} ,$$

where $u_{eq} = J_{n} \left[ {c\left( {\omega_{m}^{*} - \omega_{m} } \right) + \dot{\omega }_{m}^{*} } \right],\quad u_{r} = J_{n} \int_{0}^{t} {\chi (\tau )d\tau }$. $\chi (t)$ needs to be designed according to the stability, rapidity and steady-state performance of the system, which is the core part of the whole SMC.

Substituting Eq. (8) into Eq. (7), we obtain:

9 $$s = - \int_{0}^{t} {\chi (\tau )d\tau } - d(t).$$

The derivative of $s$ with respect to time is:

10 $$\dot{s} = - \chi (t) - \dot{d}(t).$$

$\chi (t)$ can be designed as follows:

11 $$\chi (t) = \varepsilon \cdot sign\left( s \right) + k \cdot s.$$

Considering the alternative Lyapunov function $V = 1/2 \cdot s^{2}$. Hence, the time derivative can be obtained:

12 $$\begin{aligned} s\dot{s} & = s[ - \chi (t) - \dot{d}(t)] \\ & = s[ - \varepsilon \cdot sign\left( s \right) - k \cdot s - \dot{d}(t)] \\ & \le - k \cdot s^{2} - [\varepsilon - |\dot{d}(t)|]|s|. \\ \end{aligned}$$

Once $\varepsilon > |\dot{d}(t)|$, $s\dot{s} \le 0$. According to Lyapunov and LaSalle’s invariance theory^22,23^, the sliding variable can reach sliding surface in finite time and the stability of full-order SMC design can be verified.

However, we still notice that the boundary of the $|\dot{d}(t)|$ are hard to determined. In practice situation, $\varepsilon$ is set large enough to satisfy $s\dot{s} \le 0$ and guarantee the stability. Although in full-order SMC design, the discontinuous term is integrated to obtain the corresponding control quantity, it still introduces unavoidable chattering.

### Switching gain adaptive law

The unknown uncertainty of the inner and external disturbance is handled by the online switching gain adaptive law to reduce the chattering phenomenon. The control law can be designed:

13 $$\left\{ {\begin{array}{*{20}c} {\chi \left( t \right) = K_{1} sign\left( s \right) + K_{2} s } \\ {\dot{K}_{1} = \left\{ {\begin{array}{*{20}c} {\overline{K}|s|sign\left( {|s| - \varepsilon } \right),K_{1} > \mu } \\ {\mu ,K_{1} \le \mu {\mkern 1mu} } \\ \end{array} } \right.,} \\ \end{array} } \right.$$

where $K_{1} \left( 0 \right),\overline{K},\mu ,\varepsilon$ are small positive number. According to reference^24^, $K_{1}$ has maximum value.

The alternative Lyapunov function can be selected as:

14 $$V = \frac{1}{2}s^{2} + \frac{1}{2\gamma }\left( {K_{1} - K_{1}^{*} } \right)^{2} .$$

Considering Eqs. (12) and (13), the time derivative of the alternative Lyapunov function is:

15 $$\begin{aligned} \dot{V} & = s\dot{s} + \frac{1}{\gamma }\left( {K_{1} - K_{1}^{*} } \right)\dot{K}_{1} {\mkern 1mu} {\mkern 1mu} \\ & = s\left[ { - K_{1} sign\left( s \right) - K_{2} s - \dot{d}\left( t \right)} \right] + \frac{{\overline{K}}}{\gamma }\left( {K_{1} - K_{1}^{*} } \right)|s|sign\left( {|s| - \varepsilon } \right){\mkern 1mu} {\mkern 1mu} \\ & \le - K_{2} s^{2} - K_{1} |s| + |s|\dot{d}\left( t \right) + \frac{{\overline{K}}}{\gamma }\left( {K_{1} - K_{1}^{*} } \right)|s|sign\left( {|s| - \varepsilon } \right){\mkern 1mu} {\mkern 1mu} \\ & = - K_{2} s^{2} - K_{1}^{*} |s| + |s|\dot{d}\left( t \right) - \left( {K_{1} - K_{1}^{*} } \right)|s| + \frac{{\overline{K}}}{\gamma }\left( {K_{1} - K_{1}^{*} } \right)|s|sign\left( {|s| - \varepsilon } \right){\mkern 1mu} {\mkern 1mu} \\ & = - K_{2} s^{2} - \left[ {K_{1}^{*} - \dot{d}\left( t \right)} \right]|s| + \left( {K_{1} - K_{1}^{*} } \right)\left[ { - |s| + \frac{{\overline{K}}}{\gamma }|s|sign\left( {|s| - \varepsilon } \right)} \right]. \\ \end{aligned}$$

By introducing the coefficient $\beta_{1}$ > 0, it can be seen from reference^24^ that there always exists $K_{1}^{*}$ > 0, so that from time *t* > 0, there is always $K_{1} < K_{1}^{*}$ and $K_{1}^{*} > \dot{d}\left( t \right)$. Moreover, this will lead to:

16 $$\begin{aligned} \dot{V} & \le - K_{2} s^{2} - \left[ {K_{1}^{*} - \dot{d}\left( t \right)} \right]|s| + \left( {K_{1} - K_{1}^{*} } \right)\left[ { - |s| + \frac{{\overline{K}}}{\gamma }|s|sign\left( {|s| - \varepsilon } \right)} \right]{\mkern 1mu} {\mkern 1mu} \\ & \quad + \beta_{1} |K_{1} - K_{1}^{*} | - \beta_{1} |K_{1} - K_{1}^{*} |{\mkern 1mu} {\mkern 1mu} \\ & \le - K_{2} s^{2} - \underbrace {{\left[ {K_{1}^{*} - \dot{d}\left( t \right)} \right]}}_{{\beta_{2} > 0{\mkern 1mu} }}|s| - \beta_{1} |K_{1} - K_{1}^{*} |{\mkern 1mu} {\mkern 1mu} {\mkern 1mu} \\ & \quad - \underbrace {{|K_{1} - K_{1}^{*} |\left[ { - |s| + \frac{{\overline{K}}}{\gamma }|s|sign\left( {|s| - \varepsilon } \right) - \beta_{1} } \right]}}_{{\delta {\mkern 1mu} }}{\mkern 1mu} {\mkern 1mu} \\ & \le - \beta_{2} |s| - \beta_{1} |K_{1} - K_{1}^{*} | - \delta \\ & = - \beta_{2} \cdot \sqrt 2 \frac{|s|}{{\sqrt 2 }} - \beta_{1} \cdot \sqrt {2\gamma } \frac{{|K_{1} - K_{1}^{*} |}}{{\sqrt {2\gamma } }} - \delta {\mkern 1mu} {\mkern 1mu} \\ & \le - \underbrace {{\min \left\{ {\beta_{1} \sqrt 2 ,\beta_{2} \sqrt {2\gamma } } \right\}}}_{{\beta_{\min } }}\left( {\frac{|s|}{{\sqrt 2 }} + \frac{{|K_{1} - K_{1}^{*} |}}{{\sqrt {2\gamma } }}} \right) - \delta {\mkern 1mu} {\mkern 1mu} {\mkern 1mu} {\mkern 1mu} \\ & \le - \beta_{\min } \cdot V^{1/2} - \delta . \\ \end{aligned}$$

The positive and negative of $\delta$ is unknown. The following two conditions are discussed.

① When $$|s| > \varepsilon$$, $$\delta > 0$$:
  $$\begin{gathered} - |s| + \frac{{\overline{K}}}{\gamma }|s| - \beta_{1} > 0 \hfill \\ \Rightarrow \gamma < \frac{{\overline{K}|s|}}{{|s| + \beta_{1} }}. \hfill \\ \end{gathered}$$

Because $\dot{V} \le - \beta_{\min } \cdot V^{1/2} - \delta \le - \beta_{\min } \cdot V^{1/2}$, the system is stable.

② When $|s| < \varepsilon$, $\delta$ may be negative, which may also lead to $\dot{V} > 0$.

Since the system may not to converge, the value of $|s|$ will increase. Once $|s|$ is larger than $\varepsilon$, the stability of system will be determined by condition ①. Thus, the stability of the adaptive full-order SMC is verified.

### Extend state observer design

When the simple sliding mode controller and sliding mode observer are used to control the motor, the chattering and phase delay cannot be avoided due to the large switching gain needed to overcome the large system disturbance. Therefore, the selection and improvement of the controller and disturbance observer are particularly important.

To further reduce the chattering of speed loop, the extend state observer (ESO) is used to estimate the total disturbance which is fed back as the feedforward compensation. Even though the disturbance may be estimated inaccurately, the total disturbance can be compensated partly, which can reduce the switching gain and thus, reduce the chattering.

According to Eq. (2), the linear second-order ESO^25^ can be designed as follow equations:

17 $$\left\{ \begin{gathered} \dot{z}_{1} = z_{2} + J_{n} T_{e}^{*} + l_{1} (z_{1} - \omega_{m} ) \hfill \\ \dot{z}_{2} = l_{2} (z_{1} - \omega_{m} ). \hfill \\ \end{gathered} \right.$$

$z_{1}$, $z_{2}$ are the estimated $\omega_{m}$ and $d\left( t \right)$, respectively. $l_{1}$, $l_{2}$ are the gains of the ESO, which can be tuned as illustrated in Ref.^25^. By properly adjusting the value of $l_{1}$, $l_{2}$, the estimated states will asymptotically converge to $\omega_{m}$ and $d\left( t \right)$, respectively. The stability of the observer can be ensured by adjusting the two coefficients $l_{1}$,$l_{2}$, so that the roots of the observer characteristic equation are placed in the left half plane.

**Figure 1:**
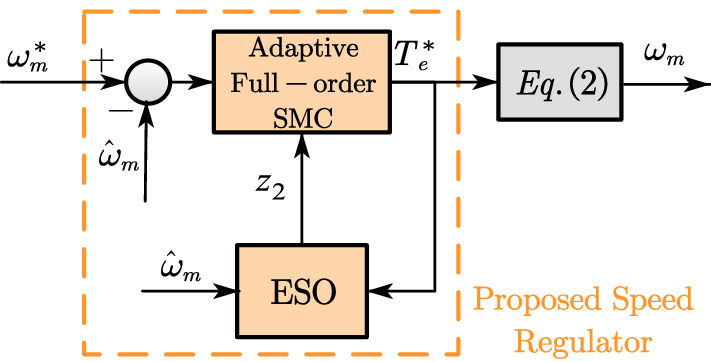
Proposed composite speed regulator for high-speed PMSM drives.

The composite speed controller is made up of two parts: the adaptive full-order SMC and the extend state observer, which is shown in Fig. 1.

The new control law is redesigned:

18 $$T_{e}^{*} = u_{eq} + u_{r} - z_{2} .$$

Substituting Eq. (15) into Eq. (7), we obtain:

19 $$s = - \int_{0}^{t} {\chi (\tau )d\tau } - [\underbrace {{d(t) - z_{2} }}_{\lambda }].$$

$\lambda$ represents the difference between real disturbance and the estimated one. The derivative of $s$ with respect to time is:

20 $$\dot{s} = - \chi (t) - \dot{\lambda }.$$

$\chi (t)$ remains in the form of Eq. (12). $\lambda$ and $\dot{\lambda }$ are bounded. The rest analysis of Lyapunov stability is totally similar to the analysis in “Switching gain adaptive law”. We only need to replace the $d(t)$ by $\lambda$. Due to the feedforward disturbance compensation, the adaptive switching gain will decrease and the chattering will be degraded.

**Figure 2:**
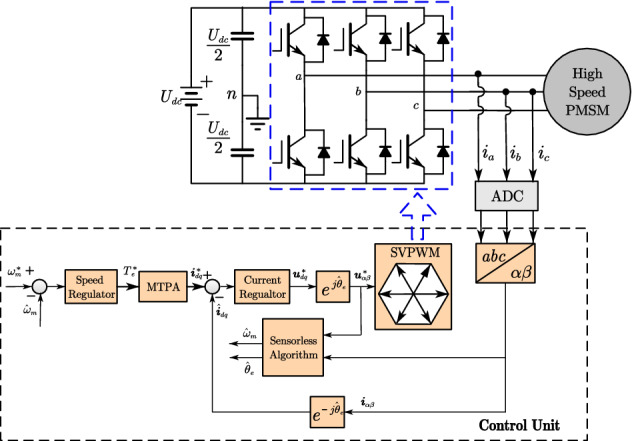
Overall block diagram of the FOC-based high-speed PMSM drives.

The overall control diagram is shown in Fig. 2. The current controller adopts the PI controllers with time-delay compensation^26^. MTPA control algorithm is used to calculate the reference current. Because the high speed PMSM is surface mounted permanent magnet synchronous motor, $i_{d}^{*} = 0$ control law is employed in control unit. The space vector pulse width modulation (SVPWM) is applied to produce the reference voltage which is generated by cascade control.

## Experimental verification

**Figure 3:**
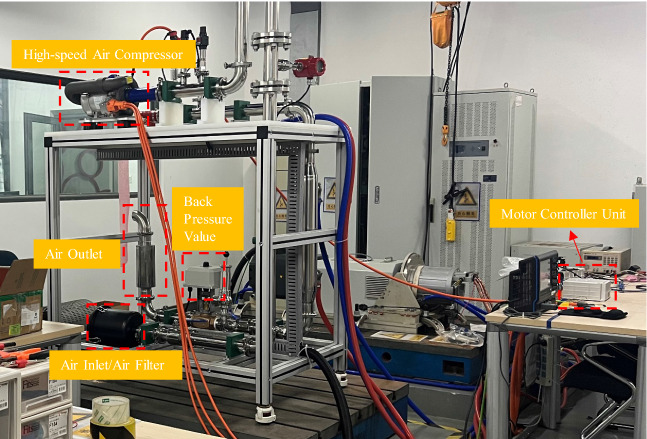
Test bench of high-speed air compressor.

In order to validate the effectiveness of the proposed composite speed regulator, the speed-tracking and anti-disturbance performance will be validated in the test bench of the high-speed air compressor, which is shown in Fig. 3. The test bench is composed of a $35\,{\text{kW}}$ high-speed air compressor, air filter/air inlet, back pressure value, air outlet, motor control unit based on the Infineon TC387 microprocessor. The load can be adjusted by the duty cycle of the back pressure value.

The parameters of the air compressor are: the stator resistance $R = 0.029\Omega$, the stator inductance $L_{s} = 104\,{\mu H}$,the permanent magnet flux $\psi_{f} = 0.026\,{\text{Wb}}$, the pole pairs $P_{n} = 1$ and the dc-link $U_{dc} = 450\,{\text{V}}$. The PWM frequency is $36\,{\text{k}}$.The idle speed of the air compressor is 30000 rpm, and the maximum speed is 80000 rpm. Thus, the proposed speed regulator is tested between 30,000 and 80,000 rpm.

**Figure 4:**
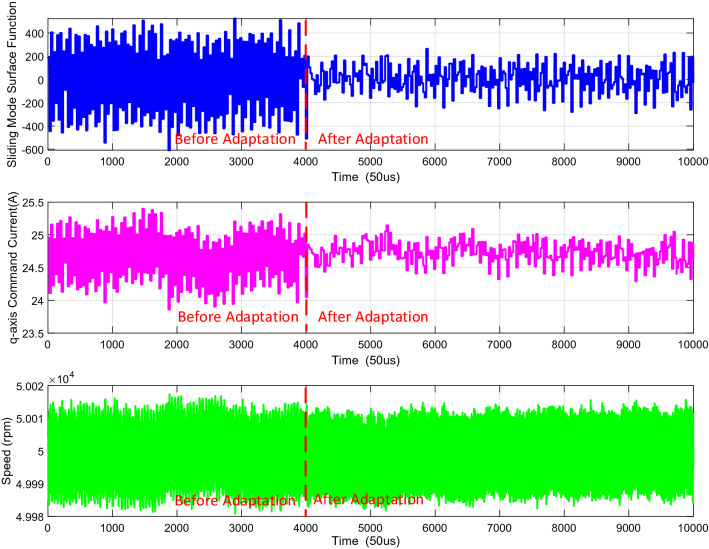
Comparison of before and after adaptation for the switching gain adaptive law at 50,000rpm.

In order to validate the effectiveness of the adaptive full-order SMC, the speed control performance before and after adaptation is compared at 50,000 rpm, which is shown in Fig. 4. It can be seen that after adopting the switching gain adaptive law, the fluctuation of the sliding mode surface function and the q-axis command current are suppressed, which means that the chattering resulting from the sliding mode switching term is reducing by means of the adjustment of the online switching gain adaptive law. It should be noted that the speed fluctuation is not affected after adaptation, even if the switching gain is reduced.

**Figure 5:**
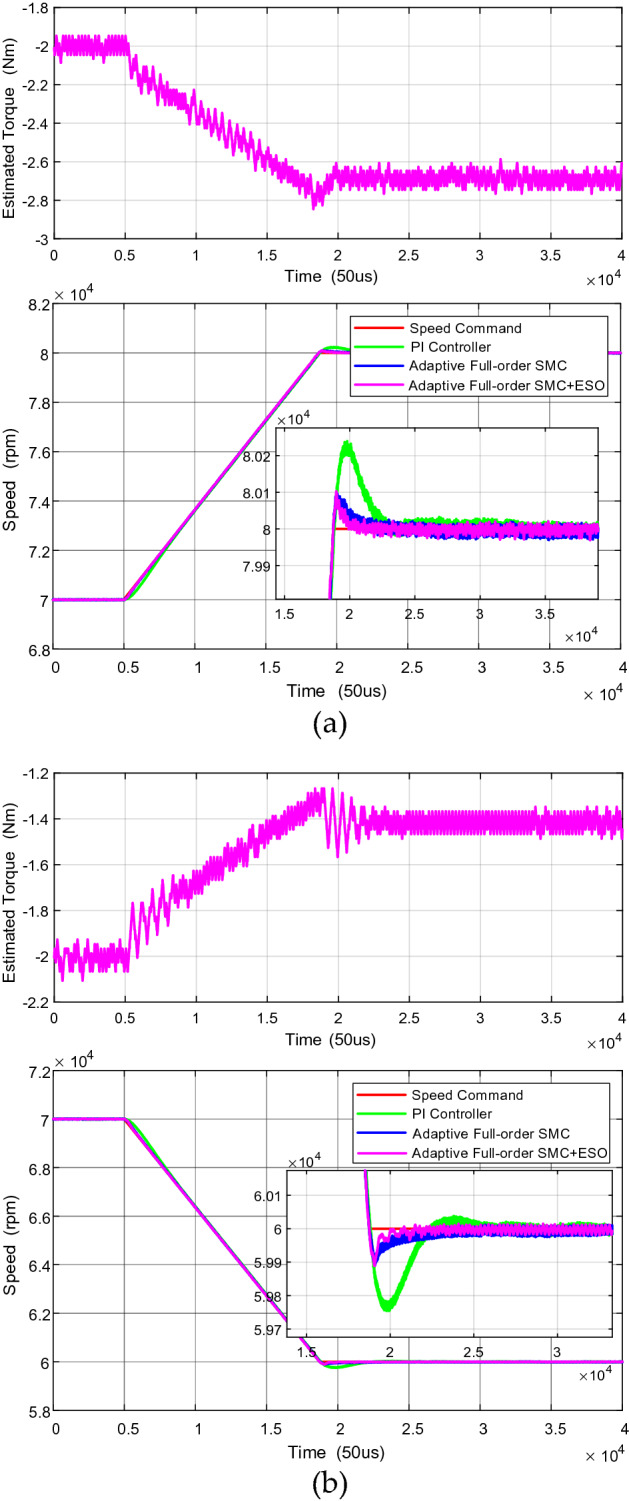
The comparison of different speed controller under the condition of (**a**) acceleration from 70,000rpm to 80,000rpm (**b**) deceleration from 70,000rpm to 60,000rpm.

Figure 5 compares the performance of different speed regulators, including PI controller, adaptive full-order SMC and the proposed composite speed regulator (adaptive full-order SMC + SMO), which is shown in Fig. 5.

As shown in Fig. 5a, during the acceleration from 70,000 to 80,000 rpm, the overshoot of PI controller is 240 rpm, while that of adaptive full-order SMC is 100 rpm. Moreover, the response time of adaptive full-order SMC is shorter. Compared with the adaptive full-order SMC, the response speed of the proposed composite speed regulator is faster due to the introduction of ESO. The response time is 0.08 s and 0.17 s, respectively. In the acceleration process, the load torque increases rapidly, since the airflow input increases with the speed rises. The ESO of the proposed composite speed regulator can estimate the load torque in real time, thus accelerating the speed response. It should be noted that, since the duty cycle of the back pressure value cannot change rapidly, the disturbance rejection performance cannot be tested by sudden loading, otherwise it will lead to the air compressor surge and damage.

Similarly, during deceleration from 70,000 to 60,000 rpm shown in Fig. 5b, it can be seen that the overshoot of PI controller is 245 rpm, while that of adaptive full-order SMC is 100 rpm. Furthermore, the speed regulator based on the PI controller oscillates when the speed reaches the steady state, resulting in longer adjustment time. Compared with the adaptive full-order SMC, the response speed of the proposed composite speed regulator is faster due to the introduction of ESO. The response time is 0.17 s and 0.36 s, respectively.

**Figure 6:**
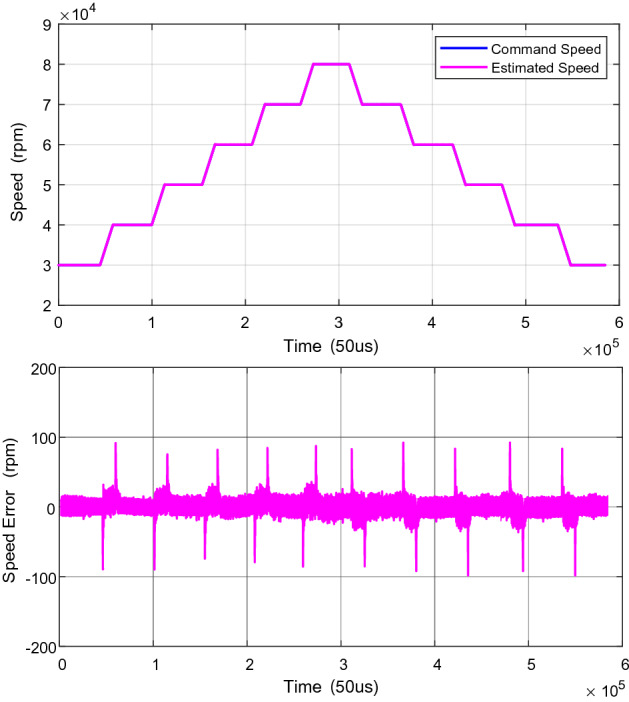
The speed response and speed error from 30,000rpm and 80,000rpm.

Figure 6 shows the speed response during acceleration and deceleration from 30,000 to 8000 rpm, and the speed error is also compared. It can be seen that the speed error is below 120 rpm and the stable speed error is below 20 rpm, which indicates the effectiveness and feasibility of the proposed composite speed regulator.

## Conclusions

In this paper, a composite speed regulator is proposed, which is made up of two parts: the adaptive full-order SMC and ESO. The adaptive full-order SMC maintains the simple structure of first-order SMC. The discontinuous terms are hidden in the integral and thus, chattering reduction is achieved. Furthermore, a switching gain adaption law is presented to minimize chattering as much as possible while ensuring the robustness of the sliding mode control. The total disturbance is estimated by the ESO and the compensation is added to the output of the controller by feed-back method, which improves the system anti-disturbance ability. Finally, the effectiveness and feasibility of the proposed speed regulator has been validated in the test bench.

## Data Availability

The datasets used and/or analysed during the current study available from the corresponding author on reasonable request.
